# Publisher Correction: An electrochemical thermal transistor

**DOI:** 10.1038/s41467-019-12471-4

**Published:** 2019-09-27

**Authors:** Aditya Sood, Feng Xiong, Shunda Chen, Haotian Wang, Daniele Selli, Jinsong Zhang, Connor J. McClellan, Jie Sun, Davide Donadio, Yi Cui, Eric Pop, Kenneth E. Goodson

**Affiliations:** 1 Department of Materials Science and Engineering, Stanford University, Stanford, CA 94305 USA; 2 Department of Mechanical Engineering, Stanford University, Stanford, CA 94305 USA; 3 Department of Electrical Engineering, Stanford University, Stanford, CA 94305 USA; 4 Department of Chemistry, University of California, Davis, CA 95616 USA; 5 Max Planck Institute for Polymer Research, Ackermannweg 10, D-55128 Mainz, Germany; 6 Ikerbasque, Basque Foundation for Science, E-48011 Bilbao, Spain; 7 Stanford Institute for Materials and Energy Science, SLAC National Accelerator Laboratory, Menlo Park, CA 94025 USA; 8 Precourt Institute for Energy, Stanford University, Stanford, CA 94305 USA; 9 Present Address: Department of Electrical and Computer Engineering, University of Pittsburgh, Pittsburgh, PA 15261 USA; 10 Present Address: Department of Chemical and Biomolecular Engineering, Rice University, Houston, TX 77005 USA; 11 Present Address: Dipartimento di Scienza dei Materiali, Universita di Milano-Bicocca, 20125 Milano, Italy; 12 Present Address: School of Chemical Engineering and Technology, Tianjin University, 300350 Tianjin, China

**Keywords:** Electrochemistry, Two-dimensional materials, Nanoscale devices, Imaging techniques, Condensed-matter physics

Correction to: *Nature Communications* 10.1038/s41467-018-06760-7, published online 30 October 2018.

The original version of this Article contained an error in Fig. 2a in which the label on the y-axis incorrectly read ‘Al/MoS_2_OiS/_2_’ rather than the correct ‘Al/MoS_2_/SiO_2_’.

Further, the original version contained an error in the last sentence of the seventh paragraph of the Discussion which incorrectly read ‘Reducing the temperature swing can have a significant effect on device reliability as the relationship between Δ*T* and the number of cycles to failure *N*_f_ is strongly non-linear^38^, *N*_f_ ~ Δ*T*^−3,5^’. The correct version states ‘Δ*T*^−3.5^’ in place of ‘Δ*T*^−3,5^’.

This has been corrected in both the PDF and HTML versions of the Article.

