# Supplementary material for: An electrochemical thermal transistor
Source: Nat Commun. 2018 Oct 30;9:4510. doi: 10.1038/s41467-018-06760-7 (PMC6207649; doi:10.1038/s41467-018-06760-7)
Supplement: Supplementary file 1 — Supplementary Information [file 41467_2018_6760_MOESM1_ESM.pdf]

Supplementary Information

An Electrochemical Thermal Transistor

Aditya Sood[1,2,*], Feng Xiong[1,3,9,*], Shunda Chen[4], Haotian Wang[1,10], Daniele Selli[5,11], Jinsong Zhang[1], Connor J. McClellan[3], Jie Sun[1,12], Davide Donadio[4,6], Yi Cui[1,7,#], Eric Pop[1,3,8,#], Kenneth E. Goodson[1,2,#]

[1]*Department of Materials Science and Engineering, Stanford University, Stanford, CA 94305, USA.* [2]*Department of Mechanical Engineering, Stanford University, Stanford, CA 94305, USA.* [3]*Department of Electrical Engineering, Stanford University, Stanford, CA 94305, USA.* [4]*Department of Chemistry, University of California, Davis, CA 95616, USA.* [5]*Max Planck Institute for Polymer Research, Ackermannweg 10, D-55128 Mainz, Germany.* [6]*Ikerbasque, Basque Foundation for Science, E-48011 Bilbao, Spain.* [7]*Stanford Institute for Materials and Energy Science, SLAC National Accelerator Laboratory, Menlo Park, CA 94025, USA.* [8]*Precourt Institute for Energy, Stanford University, Stanford, CA 94305, USA.* [9]*Present address: Department of Electrical & Computer Engineering, University of Pittsburgh, Pittsburgh, PA 15261, USA.* [10]*Present address: Department of Chemical and Biomolecular Engineering, Rice University, Houston, TX 77005, USA.* [11]*Present address: Dipartimento di Scienza dei Materiali, Università di Milano-Bicocca, 20125 Milano, Italy.* [12]*Present address: School of Chemical Engineering and Technology, Tianjin University, China.*

[*] These authors contributed equally to this work

[#] Email: yicui@stanford.edu, epop@stanford.edu, goodson@stanford.edu

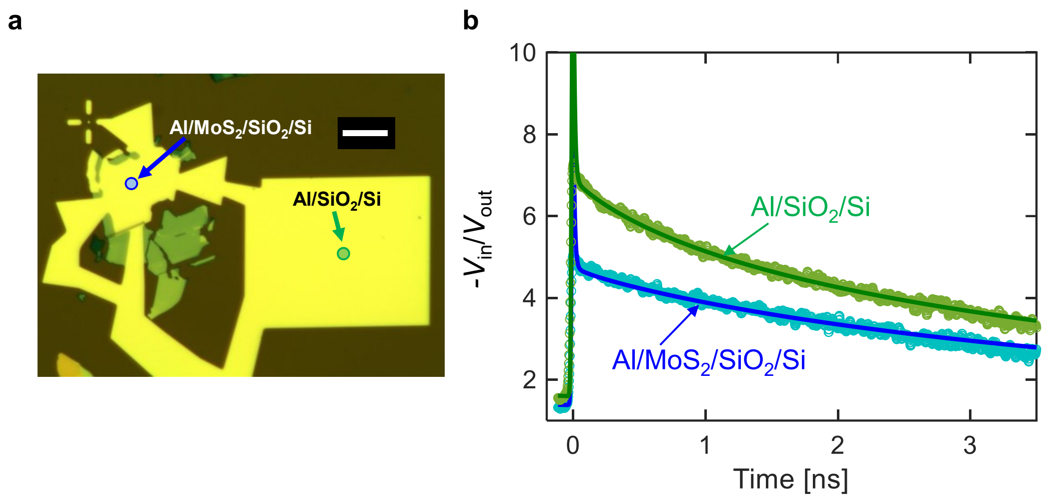

Supplementary Figure 1 | Time-domain thermoreflectance (TDTR) measurements on the Al/SiO_2/Si reference pad, and Al/MoS_2/SiO_2/Si pristine (unlithiated) MoS_2 device. a, Optical micrograph of the device, indicating the locations where measurements are made. The scale bar is 10 µm. **b**, Measured TDTR time-decay curves and best fits to the thermal model. Measurements on the reference pad give the thermal conductivity of SiO_2, $\kappa_{SiO2} = 1.4 \pm 0.1$ $Wm^{-1}K^{-1}$, and the thermal boundary conductance at the Al/SiO_2 interface, $G_{Al/SiO2} = 90 \pm 10$ $MWm^{-2}K^{-1}$, which are comparable to those reported in literature[1], validating the accuracy of our setup and analysis methodology. Measurements on the MoS_2 region give the effective cross-plane thermal conductance G, such that $1/G$ is the series sum of the Al/MoS_2 and MoS_2/SiO_2 interfacial resistances and the MoS_2 volumetric resistance. We extract a value of $G = 15 \pm 2$ $MWm^{-2}K^{-1}$. The error bars are based on uncertainties in the Al thickness t_{Al} (± 1 nm), laser spot size w_0 (± 2 %), SiO_2 conductivity κ_{SiO2} (± 0.1 $Wm^{-1}K^{-1}$), and the MoS_2 in-plane conductivity $\kappa_{r,MoS2}$ (which we vary in the range[2,3] 45 – 85 $Wm^{-1}K^{-1}$). Relatively large uncertainties in $\kappa_{r,MoS2}$ lead to only ~2 % errors in G. We also include errors resulting from uncertainties in setting the reference phase angle Φ on the lock-in amplifier. This is estimated by dividing the root-mean-square noise in the out-of-phase voltage (V_{out}) averaged between -100 to +100 ps by the jump in the in-phase voltage (V_{in}) at zero delay time; we estimate $\delta\Phi \approx 3$ mrad.

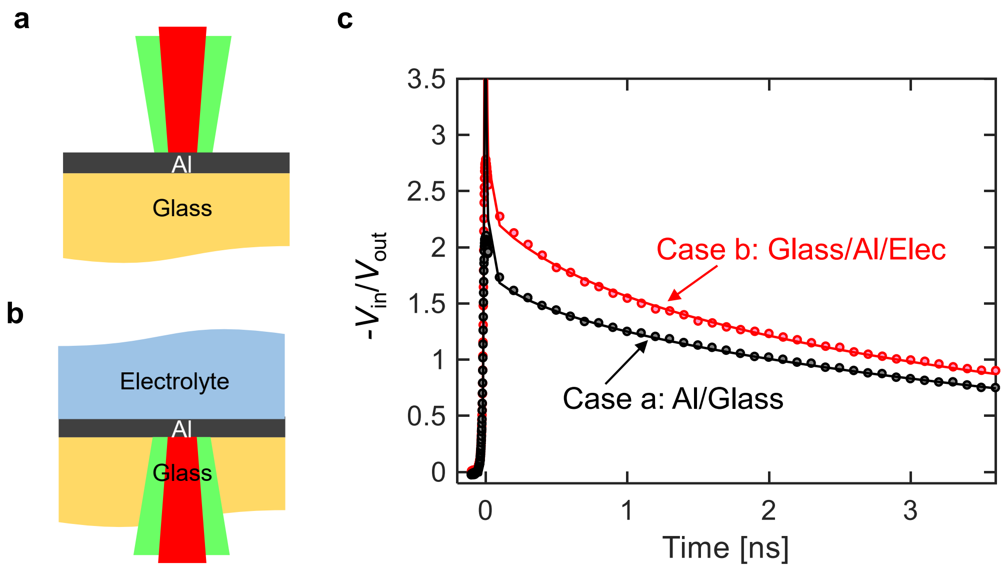

Supplementary Figure 2 | TDTR measurements of the thermophysical properties of the liquid electrolyte. **a**, Sample configuration for top-side measurements of the thermal conductivity (κ_{glass}) of the transparent fused silica (glass) substrate is: Al/glass. The Al transducer layer is ~80 nm thick. We use laser spot sizes (1/e^2 diameters) of 9.7 ± 0.2 μm and 6.0 ± 0.1 μm for pump and probe beams, respectively, and a pump modulation frequency of 10 MHz for these measurements; the measured κ_{glass} = 1.24 ± 0.1 $Wm^{-1}K^{-1}$. **b**, Sample configuration for through-substrate measurements is: glass/Al/electrolyte. A few drops of the liquid electrolyte are placed on top of the Al transducer, and encapsulated using a cover slip and epoxy sealant. We make TDTR measurements through the transparent glass (with measured conductivity κ_{glass}) and extract the thermal conductivity (κ_{elec}), volumetric specific heat (C_{elec}) of the liquid, and thermal boundary conductance at the Al/electrolyte interface ($G_{Al/elec}$); the measured κ_{elec} = 0.25 ± 0.04 $Wm^{-1}K^{-1}$ and C_{elec} = 2.4 ± 0.1 $Jcm^{-3}K^{-1}$. Error bars are based on uncertainties in t_{Al} (± 1 nm), w_0 (± 2 %) and κ_{glass} (± 0.1 $Wm^{-1}K^{-1}$). As the measurements are not very sensitive to $G_{Al/elec}$, we find that a range of values fit the data reasonably well, between 60 to 100 $MWm^{-2}K^{-1}$. These are within the range of values reported in literature for interfaces between metals and liquids[4]. **c**, TDTR time-decay curves and best fits to the thermal model corresponding to measurements in **a** and **b**.

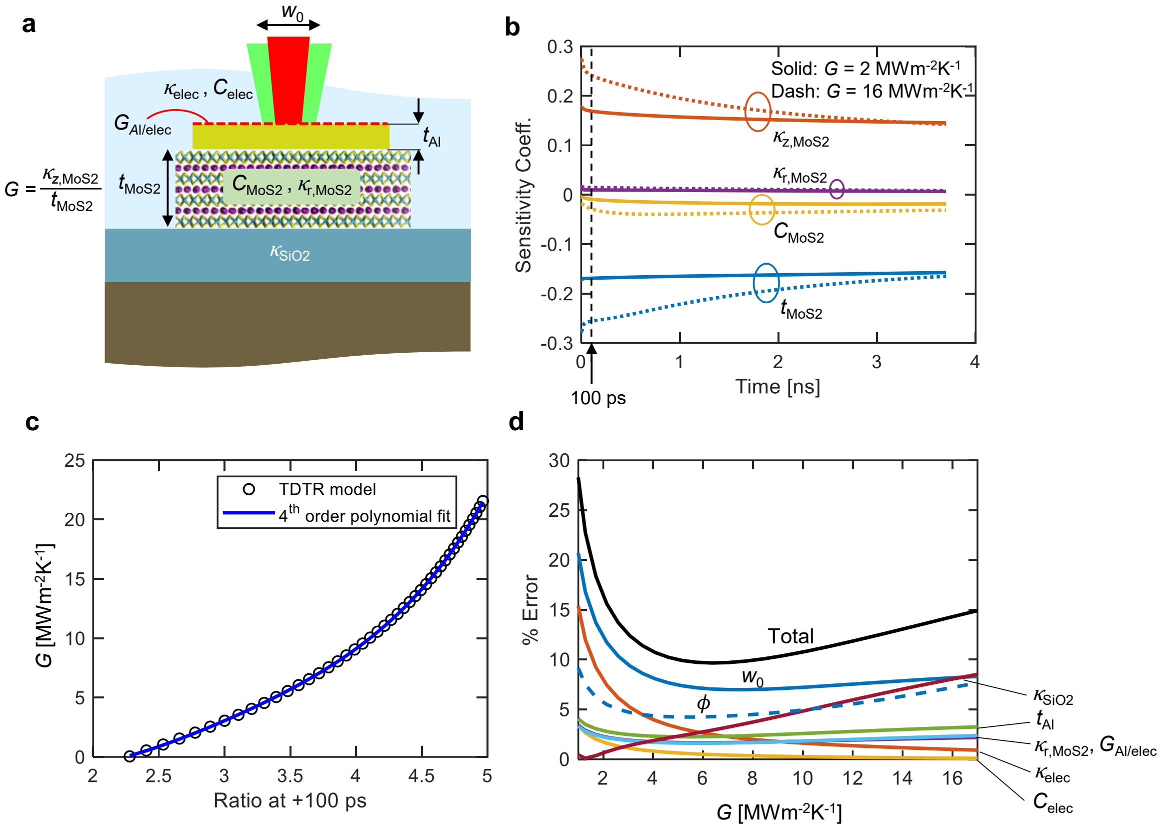

Supplementary Figure 3 | TDTR sensitivity, signal conversion and error propagation analyses for operando measurements. a, Cross-sectional schematic of the multilayer stack showing the important thermophysical properties of interest. **b**, Plot of TDTR sensitivity coefficients, calculated by dividing the fractional change in the ratio (= $-V_{in}/V_{out}$) signal by the fractional perturbation in the thermophysical parameter. Analyses presented in this figure correspond to operando measurements on a 10 nm thick device. We analyze the sensitivity to four parameters here, namely, MoS_2 thickness, cross-plane thermal conductivity, heat capacity, and in-plane thermal conductivity. The solid and dotted lines correspond to effective cross-plane conductance values of $G = 2$ MWm^{-2}K^{-1} and 16 MWm^{-2}K^{-1}, respectively. The arrow indicates the +100 ps time-delay point, where all operando temporal, and both operando and ex situ spatial mapping measurements are taken. **c**, Transfer function relating the TDTR ratio signal at +100 ps to the cross-plane thermal conductance G. Also shown is a fit to a fourth order polynomial function. The multilayer stack is: electrolyte/Al/MoS_2/SiO_2/Si. Assumed thermophysical properties for the various layers in this stack are listed in Supplementary Table 1. **d**, Error propagation analysis for operando TDTR measurements. Plotted are % errors contributed by the various sources of uncertainty. The total error is obtained by adding the individual contributing terms in quadrature (shown as the solid black line). The uncertainties in the individual parameters are: w_0 (± 2 %), κ_{elec} (± 0.04 Wm^{-1}K^{-1}), C_{elec} (± 0.1 Jcm^{-3}K^{-1}), $G_{Al/elec}$ (± 20 MWm^{-2}K^{-1}), t_{Al} (± 1 nm), $\kappa_{r,MoS2}$ (± 20 Wm^{-1}K^{-1}) and κ_{SiO2} (± 0.1 Wm^{-1}K^{-1}). Also included are uncertainties resulting from $\delta\Phi$ = 3 mrad. At low values of G when the sample is in the lithiated state, the primary contributors are κ_{elec}, w_0 and $\delta\Phi$. At higher values of G when the sample is unlithiated, the main contributors are w_0, $\delta\Phi$ and κ_{SiO2}. The total % error is calculated by adding the individual terms in quadrature: it is ± 20 % at $G = 1.6$ MWm^{-2}K^{-1}, and ± 15 % at $G = 15$ MWm^{-2}K^{-1}.

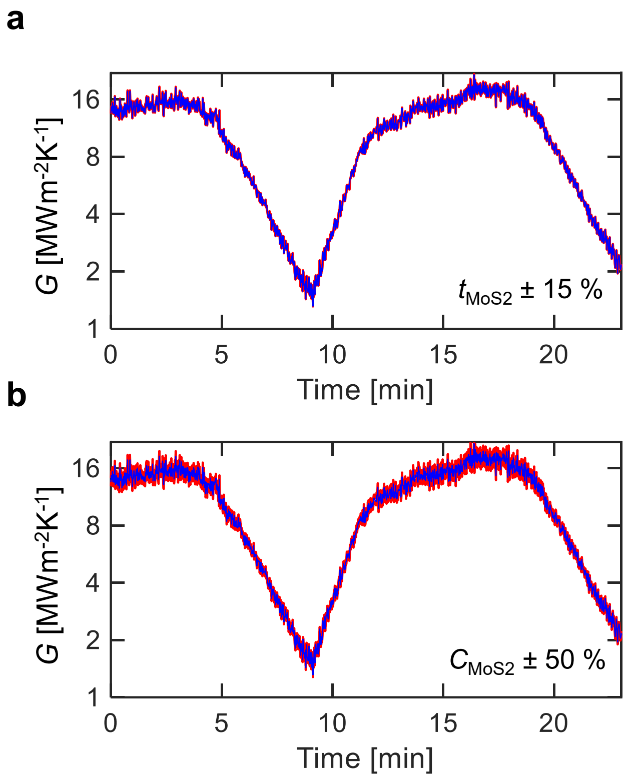

Supplementary Figure 4 | Perturbative analysis of the operando data shown in Figure 3c. a, Perturbing the thickness of the MoS_2 film (by ± 15 %) does not change the extracted thermal conductance of the device. The blue curve corresponds to the baseline case, while the red curves correspond to the thickness perturbed by ± 15 %; the curves coincide. The operando measurements are insensitive to possible lithiation-induced variations in the film thickness. **b**, Perturbing the MoS_2 heat capacity by a large amount (± 50 %) does not significantly change the extracted thermal conductance. We note that the heat capacity could increase upon lithiation, due to the additional modes in the phonon dispersion. However, any impact of these variations on the extracted thermal conductance is very small. The blue curve is the baseline case, while the red curves correspond to MoS_2 heat capacity perturbed by ± 50 % relative to the pure MoS_2 value. In the fully lithiated state (lowest G), where the heat capacity is expected to change the most, the perturbation in G is at most 2.5 %.

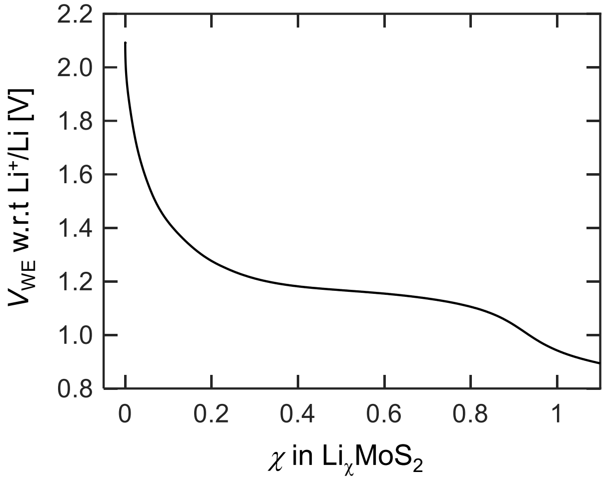

Supplementary Figure 5 | Galvanostatic discharge curve for vertically aligned MoS_2 nano-film (data from Wang et al.[5], shown here to aid discussion). The plateau at V_{WE} ~ 1.1 V indicates the 2H to 1T phase transition. At V_{WE} = 1 V, the sample reaches a lithium composition close to χ = 1. This voltage limit is used to estimate the average Li composition χ in $Li_\chi MoS_2$ in the galvanostatic experiments shown in the main text (Figure 3), for the 10 nm thick MoS_2 film. In our galvanostatic experiments, we do not use the current-time product to estimate χ since it is challenging to accurately determine the mass of an isolated MoS_2 nano-crystal, and because the fabrication process results in multiple crystals being contacted by the Al electrode (i.e. we do not know exactly how much current flows into the flake of interest). To first order, we use V_{WE} to estimate χ, relying on the fact that in a galvanostatic (i.e. constant current) experiment, the average Li composition increases linearly with time as V_{WE} is varied between the limits corresponding to χ = 0 and χ = 1 (at V_{WE} = 1 V). It is important to note that here χ is the spatially averaged Li concentration inside the device. As revealed by the thermal conductance imaging experiments shown in Figure 2, the local Li concentration is spatially non-uniform. We also note that in the galvanostatic voltage curves shown in Figure 3b, we do not see a plateau at V_{WE} = 1.1 V. This is attributed to the dominant capacitive contribution from the metal electrode, and the fast lithiation and delithiation rates (nearly 8.5 C).

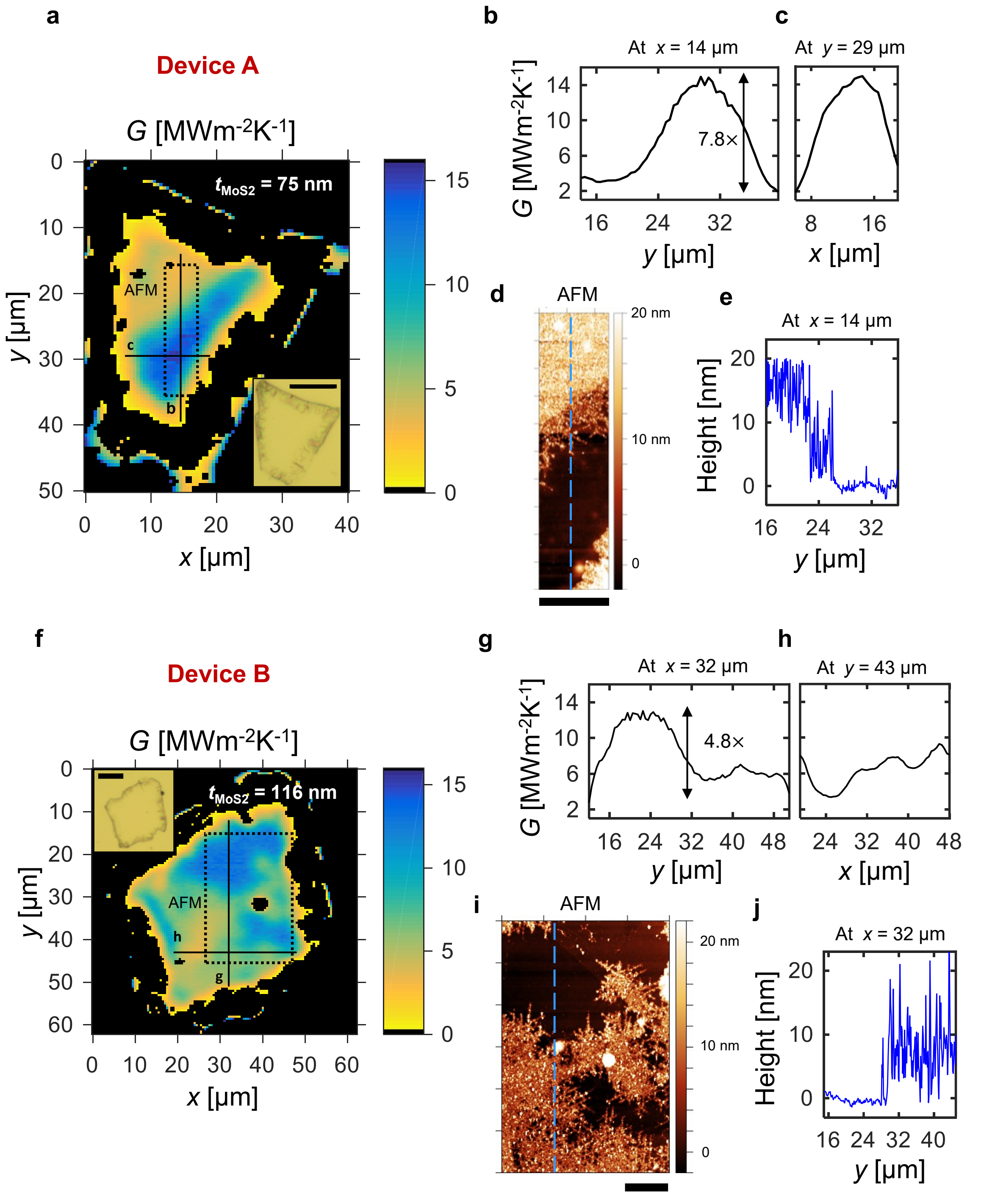

Supplementary Figure 6 | TDTR and correlative AFM data on two additional ex situ chemically lithiated devices. a-e, Device A, a 75 nm thick MoS_2 film. **f-j**, Device B, a 116 nm thick MoS_2 film. Shown in both cases are horizontal (**c**, **h**) and vertical (**b**, **g**) line scans. The maximum conductance contrast is 7.8× and 4.8× for device A and B, respectively. In both cases, AFM scans shows a clear correlation between topography and thermal conductance (**d**, **i**). The smooth unlithiated region has a higher conductance as compared to the rough lithiated region, which is also thicker on average (line scans in **e**, **j**). Scale bars in the optical micrographs (**a** and **f**) are 20 µm, and in the AFMs (**d** and **i**) are 5 µm.

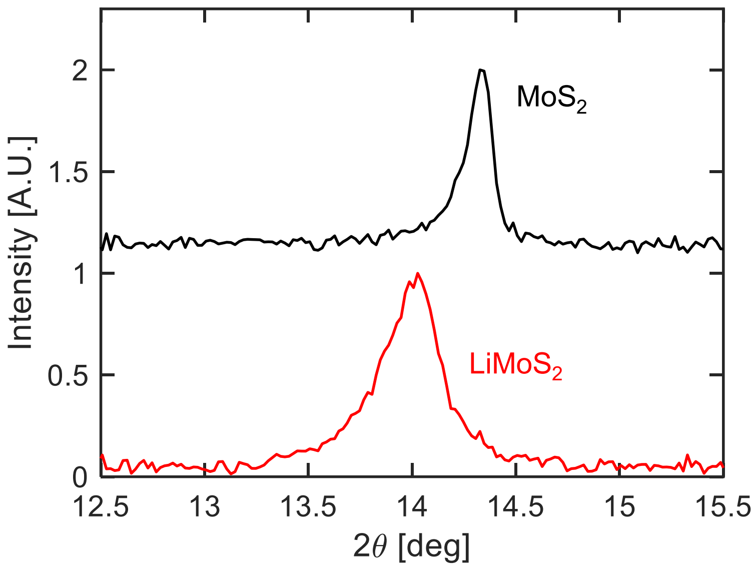

Supplementary Figure 7 | X-ray diffraction (XRD) measurements of MoS_2 and $LiMoS_2$ nano-powders. The (002) peaks corresponding to the out-of-plane lattice constant are located at 2θ = 14.34^0 and 14.02^0, for the unlithiated and lithiated samples, respectively, corresponding to c-axis lattice constants of 6.17 Å and 6.31 Å. This gives an estimated expansion of approximately 2.3 % along the c-axis due to Li intercalation. These samples were prepared by mixing MoS_2 particles (Sigma-Aldrich) with super P, coating the resulting slurry onto Al foil (cathode), and constructing an electrochemical cell with Li metal (anode), and 1 M $LiPF_6$ in EC/DEC as the electrolyte. Constant current was used to intercalate Li into MoS_2, and stopped at a voltage of 0.9 V vs Li^+/Li. The MoS_2 electrode was taken out from the pouch cell inside the glovebox and sealed by a Kapton tape for XRD measurements; the unlithiated MoS_2 was also covered by Kapton tape for comparison.

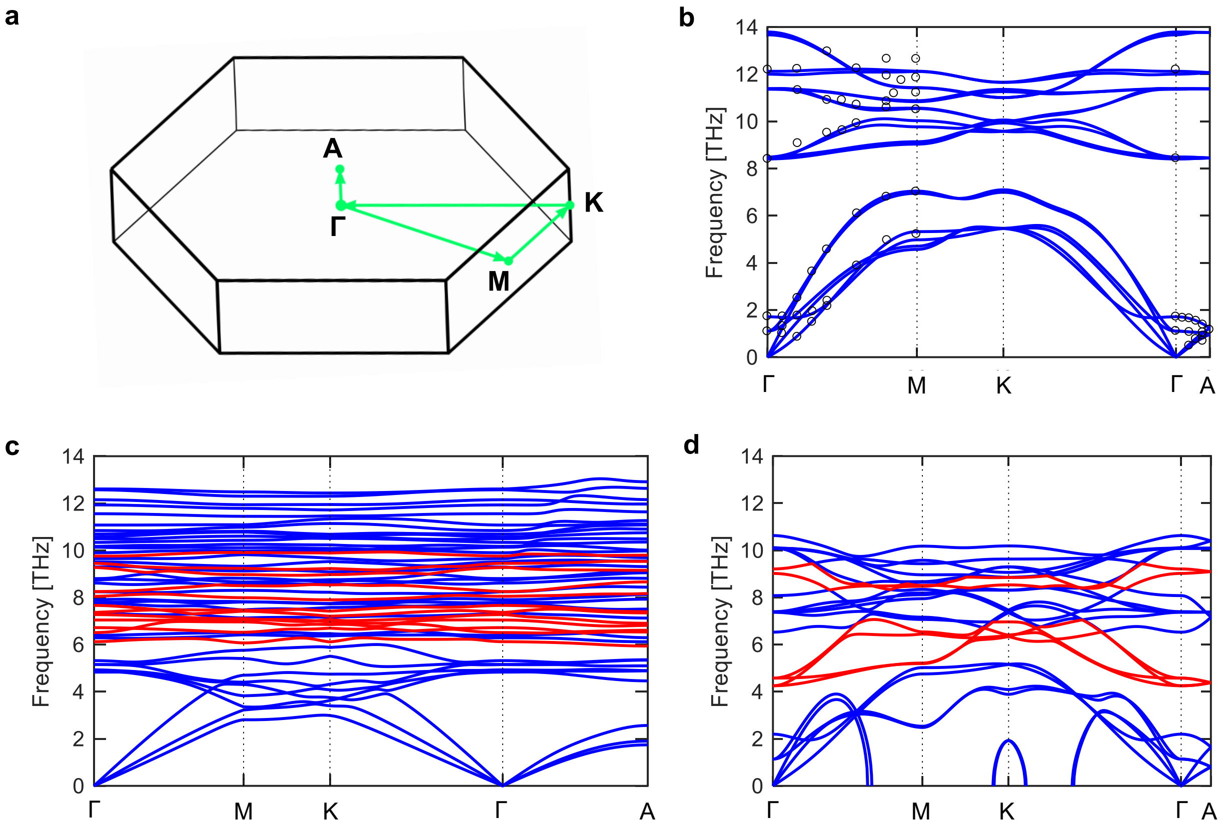

Supplementary Figure 8 | First principles DFT-LDA (density functional theory-local density approximation) phonon dispersion curves for the full Brillouin zone shown along high symmetry directions Γ-M-K-Γ-A. a, Schematic of Brillouin zone, showing the high-symmetry points along which the phonon dispersion curves have been plotted. **b**, Pure 2H-MoS_2. Black circles are neutron diffraction data for a bulk MoS_2 crystal from Wakabayashi et al.[6] showing good agreement with the calculations. **c**, 1T-Li_1MoS_2 and **d**, 2H-Li_1MoS_2, where modes are color-coded depending on whether they are MoS_2-like (blue) or Li-like (red). Soft acoustic in-plane modes occur in the thermodynamically unstable 2H lithiated phase. However, the cross-plane modes, which are most relevant to our thermal transport calculations, exhibit real eigenvalues.

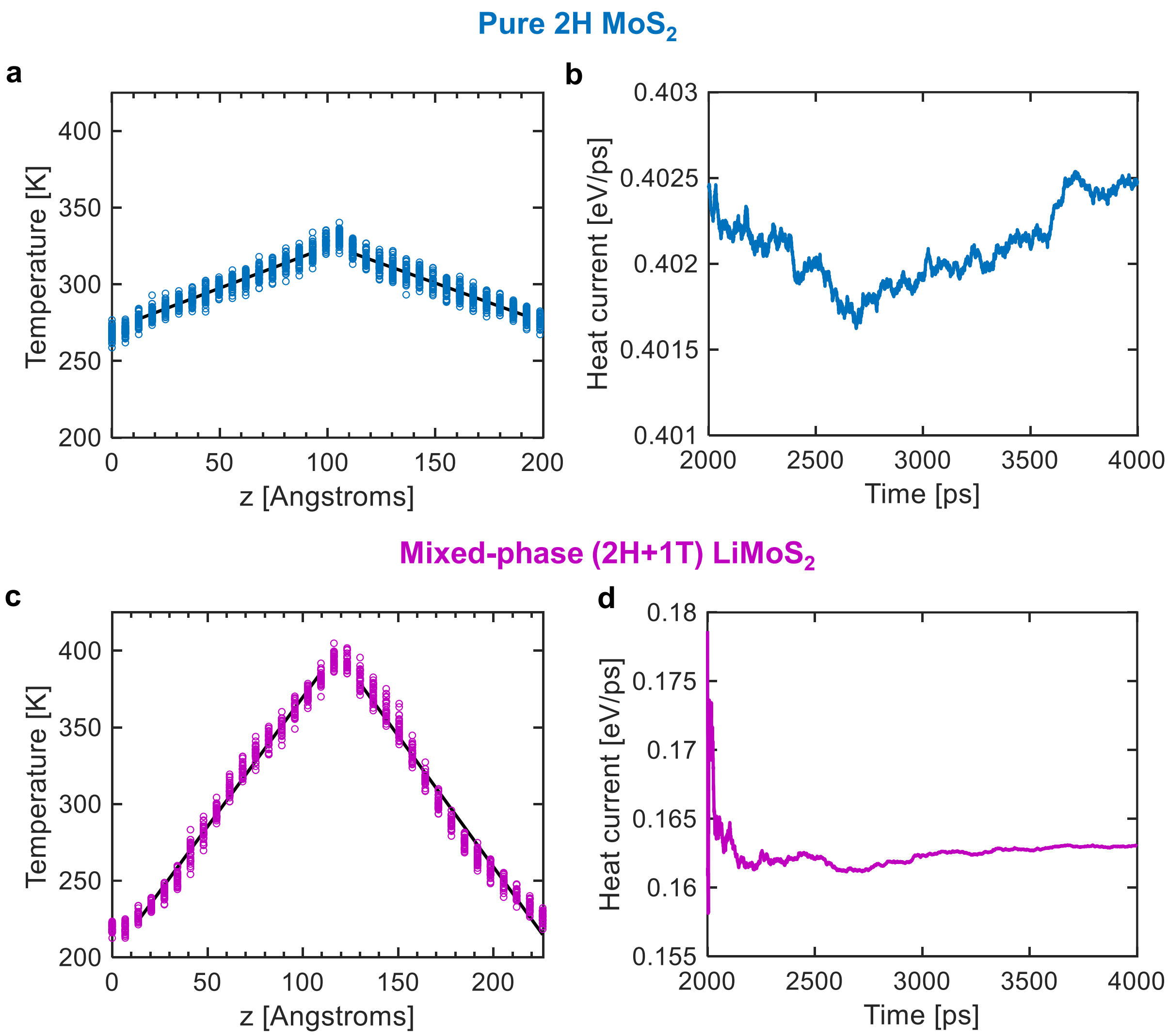

Supplementary Figure 9 | Molecular Dynamics snapshots. a, Temperature profile and **b**, Stationary heat current for the simulation of a 10 nm thick, pure (unlithiated) 2H-MoS_2 film. **c**, Temperature profile and **d**, Stationary heat current for the simulation of a 10 nm thick mixed-phase system consisting of {4, 3, 4, 2, 4} layers of {2H, 1T, 2H, 1T, 2H}, corresponding to the snapshot shown in Figure 5f.

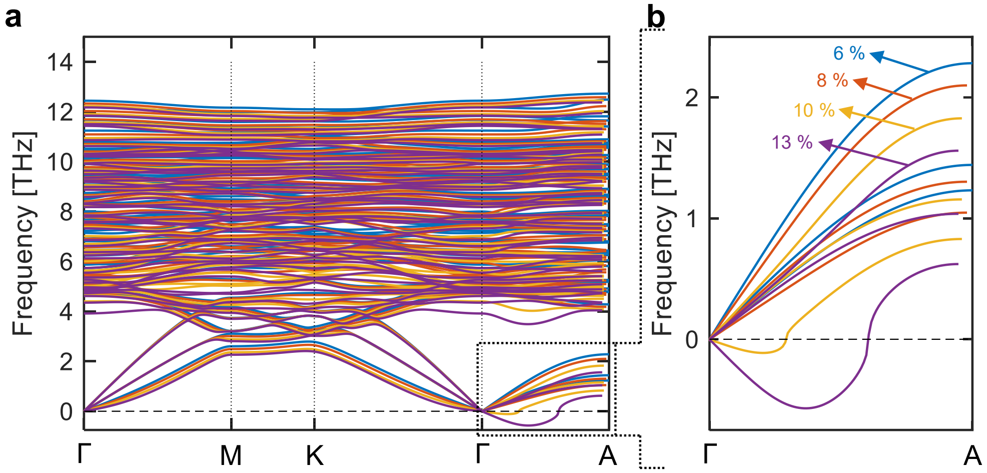

Supplementary Figure 10 | Stability of strained 1T-Li_1MoS_2. a. Ab initio phonon dispersion calculations of 1T-Li_1MoS_2 under increasing c-axis tensile strain (6, 8, 10 and 13 %, relative to unlithiated 2H-MoS_2). **b**. Zoom-in of the acoustic branches along the cross-plane direction (Γ-A). Phonon frequencies become negative for strains > 8 %, indicating structural instability.

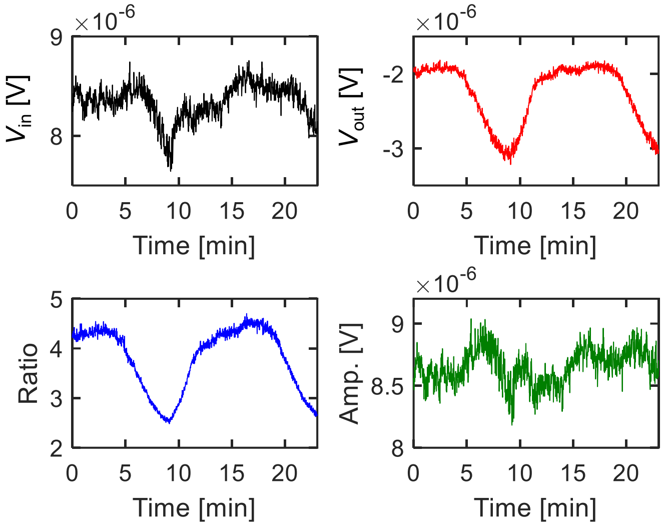

Supplementary Figure 11 | Raw TDTR data corresponding to the galvanostatic thermal conductance modulation experiment shown in Figure 3. This figure plots V_{in}, V_{out}, ratio (=-V_{in}/V_{out}) and amplitude (= $[V_{in}^2 + V_{out}^2]^{0.5}$) data at +100 ps time delay as a function of intercalation time. At this short time delay, V_{in} is relatively less sensitive to G as compared to V_{out}[7]. We see that V_{in} changes by ~8 %, while V_{out} changes by ~63 % over one electrochemical cycle.

Parameter	Value	Source
$1/e^2$ spot dia. (pump/probe) [w_0]	4.0 µm / 2.7 µm	Beam profiler, Knife edge technique[8]
Electrolyte conductivity [κ_{elec}]	0.25 $Wm^{-1}K^{-1}$	TDTR on: *glass/Al/electrolyte*
Electrolyte specific heat [C_{elec}]	2.4 $Jcm^{-3}K^{-1}$	TDTR on: *glass/Al/electrolyte*
TBC: Elec / Al [$G_{Al/elec}$]	60 – 100 $MWm^{-2}K^{-1}$	TDTR on: *glass/Al/electrolyte*
Al thickness [t_{Al}]	79.7 nm	AFM
Al conductivity [κ_{Al}]	160 $Wm^{-1}K^{-1}$	In-plane electrical + WF law
Al specific heat [C_{Al}]	2.44 $Jcm^{-3}K^{-1}$	Literature[9]
TBC: Al / MoS_2	∞	-
MoS_2 thickness [t_{MoS2}]	9.8 nm	AFM
MoS_2 in-plane conductivity [$\kappa_{r,MoS2}$]	45 – 85 $Wm^{-1}K^{-1}$	Literature[2,3]
MoS_2 specific heat [C_{MoS2}]	1.89 $Jcm^{-3}K^{-1}$	Literature[10]
TBC: MoS_2 / SiO_2	∞	-
SiO_2 thickness [t_{SiO2}]	90 nm	Ellipsometry
SiO_2 conductivity [κ_{SiO2}]	1.4 $Wm^{-1}K^{-1}$	TDTR on: *Al/SiO₂/Si*
SiO_2 specific heat [C_{SiO2}]	1.62 $Jcm^{-3}K^{-1}$	Literature[11]
Si conductivity [κ_{Si}]	120 $Wm^{-1}K^{-1}$	Literature[12]
TBC: SiO_2 / Si	∞	-
Si specific heat [C_{Si}]	1.66 $Jcm^{-3}K^{-1}$	Literature[13]

Supplementary Table 1 | Thermophysical properties used in the analysis of the operando TDTR data shown in Figure 2 and 3. The multilayer stack is: electrolyte/Al/MoS_2/SiO_2/Si. TBC - thermal boundary conductance, WF - Wiedemann Franz.

(a)	Local density approximation (LDA) unit cell lattice constants			
System	*a* (Å)	*c* (Å)	*a*-axis expansion	*c*-axis expansion
2H-MoS_2	3.142	12.053	-	-
2H-Li_1MoS_2	3.147	13.706	0.16 %	13.71 %
1T-Li_1MoS_2	6.776	6.054	7.83 %	0.46 %

van der Waals density functional (vdW-DF) unit cell lattice constants				
System	*a* (Å)	*c* (Å)	*a*-axis expansion	*c*-axis expansion
2H-MoS_2	3.158	12.284	-	-
2H-Li_1MoS_2	3.174	13.844	0.50 %	12.70 %
1T-Li_1MoS_2	6.789	6.229	7.49 %	1.42 %

(b)	Parameters of the MD Lennard Jones potential			
Pure MoS_2				
		ε (eV)	σ (Å)	*Q*
Mo	Mo	1.2680967	2.7498903	0.955290
S	S	0.0052756604	3.5641044	-0.477645
S	Mo	0.42071173	2.3775319	
$LiMoS_2$				
		ε (eV)	σ (Å)	*Q*
Mo	Mo	1.2680967	2.7498903	0.744962
S	S	0.0052756604	3.5641044	-0.454885
Li	Li	0.0002208294	3.5501168	0.164809
S	Mo	0.42071173	2.3775319	
Li	Mo	0.0009126005	0.63695992	
Li	S	0.0050032260	2.5784385	

Supplementary Table 2 | Parameters pertaining to theoretical phonon calculations. a, DFT lattice constants calculated using local density approximation (LDA). Upon lithiation to Li_1MoS_2, the 2H and 1T phases show c-axis lattice expansion of about 13.7 % and 0.5 %, respectively, relative to unlithiated 2H-MoS_2. Also shown for comparison are results computed using van der Waals density functional (vdW-DF), which are similar to those computed using LDA. **b**, Parameters of the Lennard Jones empirical potential used in molecular dynamics (MD) calculations. Charges (*Q*) are given in fraction of electron charge.

Supplementary References

1. Zhu, J., Tang, D., Wang, W., Liu, J., Holub, K. W. & Yang, R. Ultrafast thermoreflectance techniques for measuring thermal conductivity and interface thermal conductance of thin films. *J. Appl. Phys.* **108,** 094315 (2010).

2. Jo, I., Pettes, M. T., Ou, E., Wu, W. & Shi, L. Basal-plane thermal conductivity of few-layer molybdenum disulfide. *Appl. Phys. Lett.* **104,** 201902 (2014).

3. Liu, J., Choi, G. M. & Cahill, D. G. Measurement of the anisotropic thermal conductivity of molybdenum disulfide by the time-resolved magneto-optic Kerr effect. *J. Appl. Phys.* **116,** 233107 (2014).

4. Park, J. & Cahill, D. G. Plasmonic sensing of heat transport at solid-liquid interfaces. *J. Phys. Chem. C* **120,** 2814–2821 (2016).

5. Wang, H., Lu, Z., Xu, S., Kong, D., Cha, J. J., Zheng, G., Hsu, P. C., Yan, K., Bradshaw, D., Prinz, F. B. & Cui, Y. Electrochemical tuning of vertically aligned MoS_2 nanofilms and its application in improving hydrogen evolution reaction. *Proc. Natl. Acad. Sci. U. S. A.* **110,** 19701–19706 (2013).

6. Wakabayashi, N., Smith, H. G. & Nicklow, R. M. Lattice dynamics of hexagonal MoS2 studied by neutron scattering. *Phys. Rev. B* **12,** 659–663 (1975).

7. Zheng, X., Cahill, D. G., Krasnochtchekov, P., Averback, R. S. & Zhao, J. C. High-throughput thermal conductivity measurements of nickel solid solutions and the applicability of the Wiedemann-Franz law. *Acta Mater.* **55,** 5177–5185 (2007).

8. Sood, A., Cho, J., Hobart, K. D., Feygelson, T. I., Pate, B. B., Asheghi, M., Cahill, D. G. & Goodson, K. E. Anisotropic and inhomogeneous thermal conduction in suspended thin-film polycrystalline diamond. *J. Appl. Phys.* **119,** 175103 (2016).

9. Buyco, E. H. & Davis, F. E. Specific heat of aluminum from zero to its melting temperature and beyond. Equation for representation of the specific heat of solids. *J. Chem. Eng. Data* **15,** 518–523 (1970).

10. Kim, J. Y., Choi, S. M., Seo, W. S. & Cho, W. S. Thermal and electronic properties of exfoliated metal chalcogenides. *Bull. Korean Chem. Soc.* **31,** 3225–3227 (2010).

11. Andersson, S. & Dzhavadov, L. Thermal conductivity and heat capacity of amorphous SiO2: pressure and volume dependence. *J. Phys. Condens. Matter* **4,** 6209–6216 (1992).

12. Regner, K. T., Sellan, D. P., Su, Z., Amon, C. H., McGaughey, A. J. H. & Malen, J. A. Broadband phonon mean free path contributions to thermal conductivity measured using frequency domain thermoreflectance. *Nat. Commun.* **4,** 1640 (2013).

13. Flubacher, P., Leadbetter, A. J. & Morrison, J. A. The heat capacity of pure silicon and germanium and properties of their vibrational frequency spectra. *Philos. Mag.* **4,** 273–294 (1959).
