# Supplementary material for: An electrochemical thermal transistor
Source: Nat Commun. 2018 Oct 30;9:4510. doi: 10.1038/s41467-018-06760-7 (PMC6207649; doi:10.1038/s41467-018-06760-7)
Supplement: Supplementary file 2 — Description of Additional Supplementary Files [file 41467_2018_6760_MOESM2_ESM.pdf]

Description of Additional Supplementary Files

File Name: Supplementary Movie 1
Description: In operando thermal conductance microscopy. Video montage of scanning TDTR images showing Li intercalation in 10 nm thick MoS₂, taken at different stages of the electrochemical cycle.
